# Percutaneous CT-guided cordotomy for pain

**DOI:** 10.3171/2020.5.FOCVID209

**Published:** 2020-10-01

**Authors:** M. Benjamin Larkin, Robert Y. North, Ashwin Viswanathan

**Affiliations:** Department of Neurosurgery, Baylor College of Medicine, Houston, Texas

**Keywords:** cancer pain, cordotomy, functional, palliative care, percutaneous

## Abstract

Cordotomy has evolved since the first open procedure by Spiller and the first percutaneous radiofrequency cordotomy by Mullan in 1965. Today, the minimally invasive, CT-guided percutaneous radiofrequency cordotomy is mostly used for the palliative management of medically intractable somatic pain related to malignancy in well-selected patients. The risk of adverse events is minimized with the use of intraoperative stimulation monitoring. This video highlights the spinal cord anatomy at the level of C1–2, the approach to patient selection, the associated risks and benefits, and, finally, the procedural setup and key steps involved in this unique neurosurgical procedure.

The video can be found here: https://youtu.be/a-0ORqy0W2o

## Transcript

This video illustrates a CT-guided percutaneous radiofrequency electrode placement into the upper cervical spinal cord through the C1–2 interspace for ablation of the anterolateral spinothalamic tracts for the treatment of cancer-related pain.^1–5^

### Rationale for the Procedure

**0:40** The spinothalamic tract transmits nociceptive signals, temperature, and nondiscriminative touch from the contralateral side of the body. There is a somatotopic organization of axons within the tract.^3,5^

**0:55** Fibers entering from rostral and caudal segments are located in the medial and lateral parts of the tract, respectively.^3,5^

**1:04** The spinothalamic tract terminates in the contralateral VPL and VPM nuclei, as well as the intralaminar or central lateral nucleus and posterior complex of the thalamus. The centrolateral nucleus is involved in the effective and motivational response to pain, while the lateral thalamic projections are more involved in the sensory and discriminative aspects of pain sensation.^3,6^

**1:28** This procedure is most commonly utilized in those patients with severe, medically intractable cancer pain who have a life expectancy of 1 to 2 years. The procedure is best suited for patients with unilateral somatic-type pain below the level of C5. Midline pain is not responsive, typically, to this procedure.^3,7^

**1:48** Malignancies most commonly treated include mesothelioma and Pancoast tumors, gastrointestinal carcinomas, and metastatic carcinoma. Rarely, it may be utilized for the treatment of noncancer pain. For example, cordotomy may be useful in the treatment of nociceptive pain related to a hip fracture in a patient who is receiving palliative care.

**2:11** Great caution should be exercised when performing a bilateral high cervical cordotomy due to the risk of Ondine’s curse. Outcomes for patients with complete deafferentation may also be worse due to centralization of the pain syndrome. Patients with large intracranial metastases are also at increased risk due to CSF egress through the C1–2 puncture. The ideal patient should have good pulmonary reserve. Though there are not reported complications in patients with impaired pulmonary function, pulmonary function tests in order to measure their FEV-1 and FVC may be considered [https://commons.wikimedia.org/wiki/File:Lungvolumes_Updated.png].

**2:49** The corticospinal tracts lie dorsal to the spinothalamic tracts, while the ventral spinal cerebellar tracks overlie them laterally. Lesions to the spinocerebellar or corticospinal tracts can lead to postoperative ataxia or weakness of the ipsilateral leg. If the lesion is too high (cranial), it is possible to cause contralateral leg weakness. Medially lies the autonomic pathways for vasomotor and genitourinary control found in the lateral horn of the gray matter. Lesions here can result in urinary incontinence and hypotension. The most important adjacent structure is the reticulospinal tract found anteriomedially to the spinothalamic tract. Damage here can result in sleep apnea and Ondine’s curse. This can also occur as the result of reticulospinal tract lesioning in the setting of impaired pulmonary function or if the only functioning lung is on the side being lesioned.^3,5^

**3:45** It can also occur within the context of a bilateral cordotomy. Bilateral cordotomy is often reserved for bilateral lower-extremity pain and involves super-selective lesioning of the superficial and dorsal portion of the spinal cord. Dysesthesias can occur in up to 5% of patients with a neuropathic component to their pain and who are long-term survivors, as well as those who receive large lesions.^3,5^

**4:11** One important, though rare, and difficult consequence of this procedure is what is called “mirror pain,” which can occur in up to 5% of patients. This is thought to be the result of bilateral pain that is unmasked as a result of the procedure, or transmission of the dorsal spinal nerve roots into bilateral spinothalamic tracts.^3,5,7^

### Description of the Setup

**4:32** To perform the procedure, you will need a CT scanner, a radiofrequency device, a 20-gauge needle, and a disposable percutaneous radiofrequency electrode.

**4:42** The procedure is done in the interventional CT suite. Thirty minutes prior to the procedure, a myelogram is performed via lumbar puncture. Afterwards, the patient is placed in the Trendelenburg position to aid in contrast distribution.

**4:56** This is done to better visualize the spinal cord. Alternatively, this can be done after placement of the cannula during the C1–2 puncture.

**5:05** The procedure is done under a local anesthetic with monitored anesthesia care. It is important to provide local anesthesia and light conscious sedation for the initial needle placement. The patient should be woken for test stimulation and lesioning. The patient is positioned supine. It is important that the head remain immobilized in a straight and neutral position within the CT gantry. The shoulders, if needed, should be pulled down to ensure access to the neck. The patient is draped and the area around the mastoid tip is prepped in a sterile fashion.

**5:37** CT image acquisition should be parallel and 0 gantry with 1- to 1.25-mm slices from the foramen magnum to the inferior portion of C2 with a wide field of vision so as to see the skin. The field of view should be narrowed to avoid dental artifact.

### Key Surgical Steps

**5:56** Prior to insertion of needle, local anesthetic with 2% lidocaine is infiltrated into the entry point. A 20-gauge spinal needle is inserted into the skin and soft tissue just inferior to the mastoid tip, less than the distance to the dura. Serial CT scans are obtained to confirm appropriate trajectory and estimate additional distance. The trajectory is adjusted as needed as the needle is advanced into the subarachnoid space. Just prior to the puncture of the dura, 2 ml of 2% lidocaine may be injected to avoid pain secondary to the C2 ganglion and dura.

**6:34** Once CSF egress is confirmed, a repeat CT scan is obtained to confirm adequate needle placement in the anterolateral quadrant of the spinal cord contralateral to the painful side.

**6:46** Because of the somatotopic arrangement of the spinothalamic tract, the needle can be positioned more anteromedially, or 2 to 3 mm anterior to the dentate ligament, for more cervical, thoracic, and arm pain. Or, more posterolaterally, 1 mm anterior to the dentate ligament, for pain located in lumbar, sacral, or leg region.^3,5^

**7:08** The RF electrode and hub is placed within the spinal needle. The radiofrequency electrode is slowly advanced while monitoring impedance. Entry into the spinal cord is signaled once there is a sudden increase to approximately 800 to 1000 ohms.

**7:24** Final needle position. At this time, the patient may experience a brief period of pain. It is not uncommon for the radiofrequency electrode to overshoot the intended target secondary to the movement of the spinal cord and the force required to penetrate the pia.

**7:37** The electrode depth may be retracted by adjusting the probe depth on the hub.

**7:43** There is a lot of variability in the location of spinal pathways, especially with anterior cortical spinal pathways, and it is therefore critical to utilize intraoperative monitoring to ensure patient safety and avoid complications.^3,5^

**7:57** Stimulation. Sensory stimulation at 100 Hz at 0.1-msec pulse width at < 0.15 V is performed to confirm appropriate placement.

**8:09** This should give the patient a sense of warm or mild pain on the opposite half of the body in the region of pain. Usually sensory stimulation can be elicited at less than 0.2 V. However, in patients with the deafferentation, higher amplitudes may be needed up to 0.5 V. Occasionally, paresthesia cannot be elicited in the area of pain, but rather will be elicited in surrounding somatotopic areas. Adjustment of the RF electrode anteroposteriorly and mediolaterally is needed to confirm optimal electrode placement. If sensory stimulation is not elicited at less than 0.5 V, the RF electrode should be repositioned more anteromedially for cervicothoracic pain or posterolaterally for lumbosacral pain.^3,5^

**8:58** Motor stimulation at 2 Hz and 0.1-msec pulse widths up to 1 V is performed, at which time there should be no motor response. Ipsilateral neck contractions are commonly seen due to current spread to the anterior horn cells and ventral rootlets. Ipsilateral motor responses at less than 1 V indicates the electrode is too close to the dorsal corticospinal tracts and warrants repositioning.^3,5^

**9:25** Ablation. Once position is verified, a thermal ablation can be performed at 80°C for 60 seconds.

**9:32** During this portion of the procedure, the patient may elevate the leg ipsilateral to the stimulation, but contralateral to the painful side in order to detect subtle changes in motor strength throughout the process. The RF electrode may be adjusted either mediolaterally or anteroposteriorly to create a second lesion at 80°C for 60 seconds. Following lesioning, the patient should not be able to differentiate sharp or dull sensation.^3,5,7^

**10:01** Conclusions. Percutaneous cordotomy is a minimally invasive option for the treatment of unilateral, intractable somatic cancer pain below the level of C5 in a patient with a 1- to 2-year life expectancy. It can be performed relatively easily in the awake patient and improves the quality of life in a well-selected patient.^5,7^

## Author Contributions

Primary surgeon: Viswanathan. Assistant surgeon: North. Editing and drafting the video and abstract: Larkin, North. Critically revising the work: all authors. Reviewed submitted version of the work: all authors. Approved the final version of the work on behalf of all authors: Larkin.
